# Comparative Analysis of Outcomes in Adult Spinal Deformity Patients with Proximal Junctional Kyphosis or Failure Initially Fused to Upper Versus Lower Thoracic Spine

**DOI:** 10.3390/jcm13247722

**Published:** 2024-12-18

**Authors:** Oluwatobi O. Onafowokan, Renaud Lafage, Peter Tretiakov, Justin S. Smith, Breton G. Line, Bassel G. Diebo, Alan H. Daniels, Jeffrey L. Gum, Themistocles S. Protopsaltis, David Kojo Hamilton, Thomas Buell, Alex Soroceanu, Justin Scheer, Robert K. Eastlack, Jeffrey P. Mullin, Gregory Mundis, Naobumi Hosogane, Mitsuru Yagi, Neel Anand, David O. Okonkwo, Michael Y. Wang, Eric O. Klineberg, Khaled M. Kebaish, Stephen Lewis, Richard Hostin, Munish Chandra Gupta, Lawrence G. Lenke, Han Jo Kim, Christopher P. Ames, Christopher I. Shaffrey, Shay Bess, Frank J. Schwab, Virginie Lafage, Douglas Burton, Peter G. Passias

**Affiliations:** 1 Duke Spine Division, Departments of Neurological and Orthopaedic Surgery, Duke School of Medicine, Durham, NC 27710, USA; 2 Department of Orthopedics, Lenox Hill Hospital, New York, NY 10075, USA; 3 Department of Neurosurgery, University of Virginia, Charlottesville, VA, USA; 4 Department of Spine Surgery, Denver International Spine Clinic, Presbyterian St. Luke’s/Rocky Mountain Hospital for Children, Denver, CO 80205, USA; 5 Department of Orthopedics, Warren Alpert Medical School, Brown University, Providence, RI 02903, USA; 6 Norton Leatherman Spine Center, Louisville, KY 40202, USA; 7 Division of Spinal Surgery, Departments of Orthopaedic and Neurosurgery, NYU Langone Medical Center, NY Spine Institute, New York, NY 10006, USA; 8 Department of Neurological Surgery, University of Pittsburgh School of Medicine, Pittsburgh, PA 15213, USA; 9 Department of Orthopaedic Surgery, University of Calgary, Calgary, AB T2N 1N4, Canada; 10 Department of Neurologic & Orthopaedic Surgery, Columbia University, New York, NY 10027, USA; 11 Department of Orthopedics, Scripps Clinic, La Jolla, CA 92037, USA; 12 Department of Neurosurgery, University of Buffalo, Getzville, NY 14068, USA; 13 Department of Orthopedic Surgery, National Defense Medical College, Tokyo 359-8513, Japan; 14 Department of Orthopedic Surgery, International University of Health and Welfare, Tochigi 831-8501, Japan; 15 Department of Orthopedic Surgery, Cedars-Sinai Spine Center, Los Angeles, CA 90048, USA; 16 Department of Neurosurgery, University of Miami, Coral Gables, FL 33146, USA; 17 Department of Orthopaedic Surgery, UT Health, Houston, TX 77030, USA; 18 Department of Orthopaedic Surgery, Johns Hopkins Medical Center, Baltimore, MD 21224, USA; 19 Division of Orthopaedic Surgery, University of Toronto, Toronto, ON M5S 1A1, Canada; 20 Department of Orthopaedic Surgery, Southwest Scoliosis Center, Dallas, TA 75243, USA; 21 Department of Orthopaedic Surgery, Washington University, St. Louis, MO 63130, USA; 22 Department of Orthopedic Surgery, Hospital for Special Surgery, New York, NY 10021, USA; 23 Department of Neurological Surgery, University of California San Francisco, San Francisco, CA 94143, USA; 24 Department of Orthopaedic Surgery, University of Kansas Medical Center, Kansas City, KS 66103, USA

**Keywords:** adult spine deformity, realignment, spine fusion, proximal junctional kyphosis, proximal junctional failure

## Abstract

**Background:** Patients with proximal junctional kyphosis (PJK) or failure (PJF) may demonstrate disparate outcomes and recovery when fused to the upper (UT) versus lower (LT) thoracic spine. Few studies have distinguished the reoperation and recovery abilities of patients with PJK or PJF when fused to the upper (UT) versus lower (LT) thoracic spine. **Methods:** Adult spine deformity patients ≥ 18 yrs with preoperative and 5-year (5Y) data fused to the sacrum/pelvis were included. The rates of PJK, PJK revision, and radiographic PJF were compared between patients with upper instrumented vertebra (UIV) in the upper thoracic spine (UT; T1-T7) and lower thoracic spine (LT; T8-L1). Mean differences were assessed via analyses of covariance, factoring in any differences between cohorts at baseline and any use of PJF prophylaxis. Backstep logistic regressions assessed predictors of achieving Smith et al.’s Best Clinical Outcomes (BCOs) and complications, controlling for similar covariates. **Results:** A total of 232 ASD patients were included (64.2 ± 10.2 years, 78% female); 36.3% were UT and 63.7% were LT. Postoperatively, the rates of PJK for UT were lower than LT at 1Y (34.6 vs. 50.4%, *p* = 0.024), 2Y (29.5 vs. 49.6% (*p* = 0.003), and 5Y (48.7 vs. 62.8%, *p* = 0.048), with comparable rates of PJF. In total, 4.0% of UT patients underwent subsequent reoperation, compared to 13.0% of LT patients (*p* = 0.025). A total of 6.0% of patients had recurrent PJK, and 3.9% had recurrent PJF (both *p* > 0.05). After reoperation, UT patients reported higher rates of improvement in the minimum clinically important difference for ODI by 2Y (*p* = 0.007) and last follow-up (*p* < 0.001). While adjusted regression revealed that, for UT patients, the minimization of construct extension was predictive of achieving BCOs by last follow-up (model *p* < 0.001), no such relationship was identified in LT patients. **Conclusions:** Patients initially fused to the lower thoracic spine demonstrate an increased incidence of PJK and lower rates of disability improvement, but are at a lessened risk of neurologic complications if reoperation is required.

## 1. Introduction

Adult spine deformity is a heterogenous pathology with the potential to cause significant disability in affected individuals [1]. Proximal junctional kyphosis (PJK) is a well-recognized complication following corrective surgery for adult spinal deformity (ASD). It refers to the development of kyphosis above the upper instrumented vertebra and is a major concern for both surgeons and patients [2,3]. PJK can lead to various clinical problems, including pain, neurological deficits, and the need for revision surgery [2,3]. Similarly, failure at the proximal junction (PJF) is another important complication associated with spinal deformity surgery. PJF refers to the occurrence of hardware failure or loss of deformity correction at the proximal junction, which often necessitate additional surgery [3,4]. Both PJK and PJF have a significant impact on patient outcomes and can compromise the success of ASD corrective procedures.

To optimize surgical strategies and improve patient outcomes, it is crucial to understand the factors associated with PJK and PJF, as well as their implications for reoperation and recovery. Previous studies have reported on the incidence and risk factors for PJK and PJF in ASD patients [3,4]. However, few studies have specifically examined the differences in outcomes based on the level of fusion in the thoracic spine.

Upper instrumented vertebra (UIV) selection during ASD surgery is subject to a myriad of factors, and there is generally an endeavor to strike a balance between creating a stable construct, reducing stress on segments outside the construct, and preserving motion in the segments adjacent to the construct [5,6,7,8,9]. The level of fusion in the thoracic spine, specifically the upper thoracic (UT) versus the lower thoracic (LT) spine, may play a significant role in the occurrence and management of PJK and PJF [10,11]. However, the existing literature lacks comprehensive comparative analyses that distinguish between the outcomes of patients with PJK and/or PJF when fused to the UT or LT spine. In this context, this study aimed to investigate the outcomes in ASD patients with UIV within the UT or LT spines, and also to examine how this influenced the development of PJK and/or PJF. We hypothesized that both cohorts of patients would demonstrate distinct patterns in outcomes.

## 2. Methods

### 2.1. Data Source and Study Design

This is a retrospective analysis of a prospectively collected, multi-center database containing ASD patients enrolled at thirteen participating centers from 2009 to 2018. Institutional Review Board approval was obtained at each participating enrollment site and all patients provided informed consent. This study was approved by the HCA-HealthONE Institutional Review Board (Denver, Colorado; Approval code: 231842-22) and is registered on ClinicalTrials.gov (NCT00738439). The inclusion criteria for the database have been detailed in previous research utilizing this dataset to investigate various aspects of ASD assessment and management [12]. The patients included in this study had undergone spine fusion from the thoracic spine to the pelvis, and were followed up for up to 5 years. Patients with an upper instrumented vertebra (UIV) above T1 were excluded.

### 2.2. Data Collection and Radiographic Assessment

Standardized data collection forms tracked patient demographics, surgical parameters, and comorbidities (via the Charlson Comorbidity Index [CCI]) beginning at the initial presentation. Health-Related Quality of Life (HRQL) metrics collected at baseline and multiple follow-up time points included the Oswestry Disability Index (ODI), EuroQol 5-Domain questionnaire (EQ5D), and Scoliosis Research Society-22r (SRS-22r). Osteoporosis was defined as a T-score of ≤−2.5 on Dual X-ray Absorptiometry (DEXA) scanning of the hip and lumbar spine. Frailty was calculated in a linear and categorical manner according to the established modified ASD frailty index (mASD-FI) [13].

Full-body plain-film radiographs in the sagittal plane were obtained in order to assess relevant radiographic alignment metrics at patients’ baseline visit and at all follow-up timepoints. Radiographic imaging was analyzed with SpineView^®^ (ENSAM, Laboratory of Biomechanics, Paris, France) [14,15,16]. The spinopelvic radiographic parameters assessed included numerous metrics commonly used in the assessment of spine deformity [17]. Pelvic incidence (PI) was measured as the angle formed between a perpendicular line to the sacral plate and a line connecting the midpoint of the sacral endplate and the center of the bicoxofemoral axis. Lumbar lordosis (LL) was measured as the angle formed between lines perpendicular to the superior endplate for the L1 and S1 vertebrae. The T1 pelvic angle (TPA) was measured as the angle formed between a line connecting the center of the bicoxofemoral [17].

### 2.3. Assessment of Sagittal Alignment

The severity of deformity severity was assessed using the Scoliosis Research Society-Schwab (SRS-Schwab) classification system for adult spinal deformity (ASD) [18]. This system is a validated method for stratifying ASD patients and classifies patients based on a description of their curve type and graded sagittal descriptors based on patients’ pelvic incidence–lumbar lordosis mismatch (PI-LL), sagittal vertical axis (SVA), and pelvic tilt (PT). The sagittal spinal shape of each patient was also analyzed using the Roussouly classification, and patients were classified by their “theoretical” and “current” Roussouly types, as previously published [19,20]. The Roussouly classification categorizes sagittal spine morphology into four types, based on the pelvic incidence. A “mismatch” between the preoperative and postoperative Roussouly type has been associated with an increased mechanical complication risk [21]. Age-adjusted alignment targets and sagittal age-adjusted score (SAAS) for sagittal correction were assessed using previously published formulas established by Lafage et al. [22]. The SAAS is a spine deformity alignment classification that determines the ideal deformity correction (or “matching”) for affected patients based on the achievement of age-adjusted targets in a combination of sagittal alignment metrics (PI-LL, PT, and TPA).

### 2.4. Proximal Junctional Kyphosis and Failure

Clinically significant proximal junctional kyphosis (PJK) was defined as a proximal junctional angle magnitude of ≤−28° and a change of ≤−20° [23]. This value is higher than the commonly cited angular value for PJK, and has been described as the threshold that is associated with a notable clinical impact on affected patients. PJF was defined as patients undergoing subsequent revision surgery due to PJK or a proximal junctional sagittal Cobb angle of ≥ 15°, in the presence or absence of evidence of vertebral body fracture, implant fracture or displacement, or disruption of the osseo-ligamentous complex [3]. The PJK/PJF prophylaxis techniques employed include modalities described and established in the literature such as cement augmentation, transverse process hooks, vertebral body tethers, percutaneous screw fixation, or any combinations (“hybrid”) of the aforementioned techniques [2,3]. Implant failure was defined as any mechanical complications involving the implanted hardware, such as the dislodgement/migration of interbody devices, screw loosening or breakage, implant pullout, or rod breakage.

In this study, we defined a “Good Clinical Outcome” (the primary outcome measure) as the absence of symptomatic proximal junctional kyphosis/proximal junctional failure (PJK/F), or mechanical complications at 5 years. Other outcome measures included a minimum clinically important difference (MCID) in the improvement of the ODI score. The minimum clinically important difference in ODI was set at 11%, as per previously published research [24].

### 2.5. Statistical Analysis

The rates of PJK, revision surgery for PJK, and PJF were assessed and compared between patients whose UIV ended in the upper thoracic spine (UT; T1-T6) and lower thoracic spine (LT; T7-L1). Baseline and peri/postoperative factors were assessed using analyses of covariance while controlling for any demographic, clinical, and deformity differences and PJK/PJF prophylaxis measures. Backstep logistic regressions assessed the predictors of achieving a “Good Clinical Outcome” (GCO), controlling for BL demographic, clinical, and deformity differences and the PJK/PJF prophylaxis measures used.

## 3. Results

### 3.1. Patient Demographics

A total of 232 patients were included. The mean age of the cohort was 64.2 ± 10.2 years, with 78% of the cohort being female. At baseline, the mean cohort BMI was recorded as 28.1 ± 5.5 kg/m^2^, and the mean Charlson Comorbidity Index (CCI) was recorded as 1.74 ± 1.71. By mASD-FI, 46.6% of patients were classified as Not Frail (NF), 37.0% were classified as Frail (F), and 16.3% were classified as Severely Frail (SF). Of the included patients, 36.3% had a UIV in the UT spine, compared to 63.7% of patients with a UIV in the LT spine (Table 1).

### 3.2. Surgical Characteristics

The surgical characteristics of the entire cohort were as follows: a mean operative time of 446.9 ± 176.6 min, mean levels fused of 11.6 ± 4.1, and a mean estimated blood loss (EBL) of 1848.2 ± 1487.1 mL. According to the Schwab osteotomy grading system, a grade 3 or higher osteotomy was performed in 67% of patients. A three-column osteotomy was performed in 18.5% of patients. In terms of surgical approach, a posterior-only approach was utilized in 65% of patients, and a combined approach was utilized in 35% of patients. There were no differences in the rates of surgical approach between both cohorts. UT patients demonstrated a greater operative time (503.5 vs. 416.7, *p* = 0.012) and mean levels fused (12.4 vs. 7.8, *p* = 0.031) [Table 2]. The mean estimated blood loss did not differ between UT and LT patients. However, LT patients demonstrated higher rates of implant failure (295 vs. 5%, *p* < 0.001).

### 3.3. Radiographic Overview

Postoperatively, the incidence of PJK for UT vs LT patients at six weeks was 35.9 vs. 42.3% (*p* = 0.357), at 1 year was 34.6 vs. 50.4% (*p* = 0.024), at 2 years was 29.5 vs. 49.6% (*p* = 0.003), and by last follow up at 5 years was 48.7 vs. 62.8% (*p* = 0.048). There were no differences in the rates of matching Roussouly type preoperatively and postoperatively between both cohorts. At 5 years postop, 57.3% of patients demonstrated the achievement of at least one age-adjusted match: PT matched (27.0%), PI–LL matched (22.8%), and SVA matched (30.8%). In terms of the SAAS parameters, the mean PI–LL offset was 11.91 ± 20.25°, mean PT offset was −2.69 ± 10.17°, and mean T1 pelvic angle (TPA) offset was 5.10 ± 12.31°. In total, 22.5% of patients were considered to be matched in the SAAS criteria postoperatively. Patients who went on to develop PJK or PJF had higher odds of demonstrating greater degrees of offset by PT (OR 2.35, 95% CI: 1.57–5.38, *p* = 0.009) and TPA (OR 3.04, 95% CI: 1.68–6.29, *p* = 0.011), and were more frequently undercorrected as per SAAS scoring (61.22% vs. 49.18%, *p* = 0.026).

### 3.4. Postoperative Radiographic Analysis

When examining radiographic alignment by the 5-year postoperative mark, improvement in at least 1 SRS-Schwab modifier was seen in 64.1% of patients. Similarly, by 5 years postop, Roussouly type match was noted in 48.0% of patients, and the achievement of age-adjusted metrics was demonstrated in 40.7% patients. However, 23.9% of patients were considered to be matched in the SAAS criteria, with undercorrection noted in 21.6% of patients. The rates of PJF were not significantly different between the UT and LT groups. In total, 4.0% of UT patients underwent subsequent reoperation, compared to 13.0% of LT patients (*p* = 0.025). A total of 6.0% of all patients had recurrent PJK postoperatively, and 3.9% had recurrent PJF by last follow-up. These rates did not differ between UT and LT patients. When reoperation was indicated, LT patients had higher mean proximal levels fused compared to their UT counterparts (4.7 vs. 2.2, *p* < 0.001).

### 3.5. Patient-Reported Outcomes and Predictors of Complications

**Figure 1:**
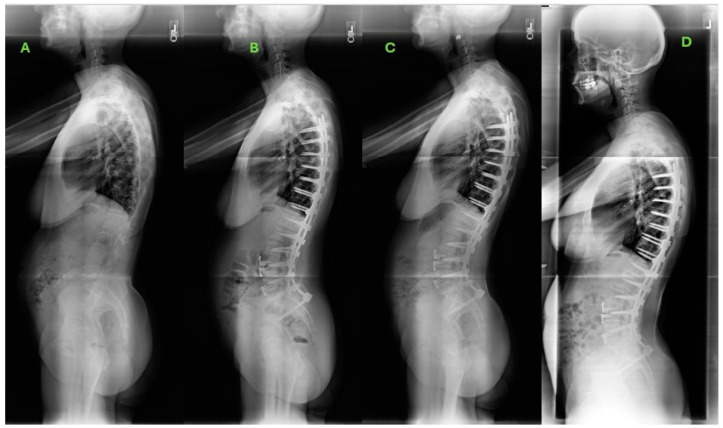
Example UT patient. A 63-year-old woman with a body mass index of 20.6 kg/m^2^ and presenting with chronic back and leg pain. She had a past medical history of gastroesophageal reflux disease and diabetes. She underwent posterior spinal fusion from T6 to pelvis and L3-S1 interbody fusion. (**A**) Baseline sagittal plain film radiograph. (**B**) Six-week postoperative sagittal radiograph. (**C**) One-year postoperative sagittal radiograph denoting mild proximal junctional kyphosis (PJK). (**D**) Five-year postoperative sagittal radiograph illustrating further progression of PJK which had also become symptomatic.

**Figure 2:**
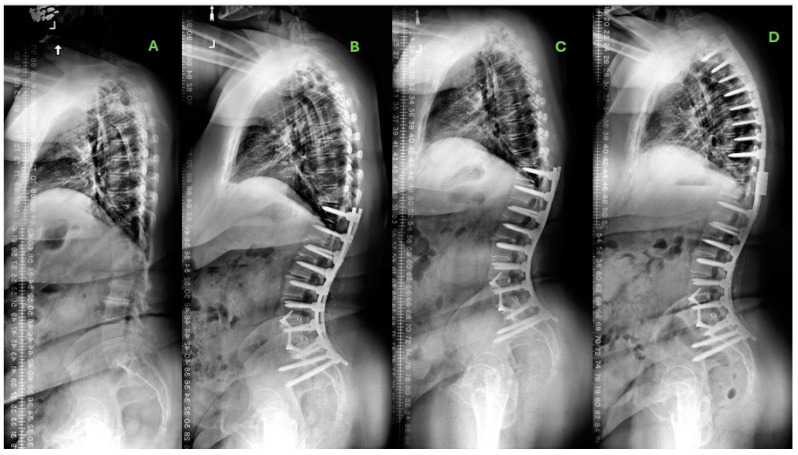
Example LT patient. A 64-year-old woman with a body mass index of 29.6 kg/m^2^ and presenting with chronic back pain. She had a past medical history of hypertension and coronary artery disease. She underwent posterior spinal fusion from T11 to pelvis and L4-S1 interbody fusion. (**A**) Baseline sagittal plain film radiograph. (**B**) Six-week postoperative sagittal radiograph. (**C**) One-year postoperative sagittal radiograph illustrating proximal junctional kyphosis (PJK) which was clinically symptomatic. The patient subsequently underwent reoperation with fusion extension proximally to T2. (**D**) Five-year postoperative sagittal radiograph.

The reoperation rates differed between both cohorts (38% UT vs. 56% LT, *p* = 0.007). Among the patients undergoing reoperation, the rates of patients undergoing reoperation for clinically significant PJK also differed significantly (31.3% UT vs. 39.8% LT, *p* = 0.018). For patients who required reoperation for PJK, UT patients reported higher rates of improvement in disability metrics by demonstrating a higher frequency of achieving the minimum clinically important difference (MCID) in ODI improvement by 2 years (78% vs. 66%, *p* = 0.007) and by 5 years (74% vs. 55%, *p* < 0.001). Multivariable logistic regression, factoring in differences in osteoporosis rates, radiographic metrics, and surgical invasiveness (using levels fused as a proxy), revealed that, for UT patients, construct extensions of ≤ two levels were an independent predictor of achieving a GCO by 5 years (OR 3.65, 95% CI: 2.74–9.33, *p* < 0.001). However, no such relationship was identified in LT patients (GCO: OR 1.58, 0.83–4.21. *p* = 0.353). Furthermore, regression analysis found that, in UT patients, non-match in SAAS at 6 weeks (OR 1.8, 95% CI 1.44–5.52, *p* < 0.001) and requiring fusion extension were significant predictors of neurological complications requiring reoperation (OR 2.73, 95% CI 2.01–11.34, *p* < 0.001). Figure 1 and Figure 2 illustrate example cases of UT and LT patients with PJK development.

## 4. Discussion

In our study, we observed significant differences in the prevalence of proximal junctional kyphosis (PJK) between patients fused to the UT versus LT spine. The rates of PJK were consistently higher in patients with fusion extending to the LT spine at various postoperative time points. This finding is consistent with previous studies reporting an increased incidence of PJK with more distal levels of fusion [25,26,27]. Biomechanical stress on the adjacent segments is believed to be higher when the upper instrumented vertebra ends in the lower thoracic region, leading to a higher risk of PJK [28]. There is notable paucity in the literature directly comparing outcomes related to the level of upper instrumented vertebra (UIV) in the thoracic spine. However, studies have trended towards more favorable outcomes in patients fused to the UT spine. Daniels et al. studied 303 patients (169 UT and 134 LT) and reported that the UT cohort (T1-T6 UIV) had significantly lower PJK rates than the LT cohort (T9-L1 UIV) [25]. Fujimori et al. reported similar findings in their study, with their UT cohort (T1-T6 UIV) also demonstrating lower PJK rates than their LT cohort (T7-T12 UIV) [26].

Interestingly, the rates of proximal junctional failure (PJF) in our study were comparable between patients fused to the UT and LT spine. This suggests that the risk of hardware failure or loss of deformity correction at the proximal junction is not significantly influenced by the level of fusion in the thoracic spine. Other factors, such as patient characteristics, surgical technique, and implant selection, may play more significant roles in the occurrence of PJF [3]. The reoperation rates differed significantly between patients fused to the UT and LT spine. Our results showed that patients with fusion ending in the LT spine had a higher reoperation rate compared to those fused to the UT spine. This finding indicates that patients initially fused to the lower thoracic spine are more likely to require additional surgery following the development of PJK or PJF. These results are consistent with previous studies reporting higher revision surgery rates in patients with more distal upper instrumented vertebra levels [27].

When assessing health-related quality of life metrics, there were no differences in scores at all timepoints. This was demonstrated even with the increased invasiveness and longer length of stay in the UT cohort. This finding has been supported in meta-analyses reporting insignificant differences in the HRQL between UT and LT patients [27,28]. However, it is worth noting that UT patients demonstrated higher rates of achieving the MCID in ODI improvement.

Our study also identified potential predictors of reoperation and achieving good outcomes based on the level of fusion. In patients fused to the UT spine, minimizing construct extension was found to be an independent predictor of achieving a GCO. This indicates that avoiding extensive fusion levels beyond the UT spine may improve the chances of achieving favorable postoperative outcomes. In contrast, no such relationship was identified in patients fused to the LT spine, suggesting that factors other than construct extension may play a more prominent role in determining outcomes in this group.

Additionally, a predictive analysis identified specific factors associated with neurological complications requiring reoperation in patients fused to the UT spine. Non-match in 6-week age-adjusted criteria and requiring fusion extension were significant predictors of such complications. This finding highlights the importance of careful patient selection and surgical planning to minimize the risk of neurological complications in UT fusion cases. Studies reporting neurological complications following ASD surgery with spinal fusion involving the thoracic spine have also reported other potential risk factors such as deformity etiology and severity, deformity angular ratio, combined (anterior and posterior) surgical approaches, undergoing high-grade osteotomies, and age [29,30].

It is important to note that, while primary fusion to the UT spine may lessen the risk of reoperation for PJK or PJF, these procedures may be limited by patient age, frailty, or physiological factors. Surgeons should carefully evaluate the individual patient’s characteristics and consider the potential benefits and limitations associated with the fusion levels in the thoracic spine.

Our study provides valuable insights into the differences in the various outcomes between patients with PJK or PJF initially fused to the UT versus LT spine. Patients initially fused to the lower thoracic spine demonstrated an increased incidence of PJK and lower rates of disability improvement, but were at a decreased risk of neurologic complications if reoperation was required. On the other hand, increasing levels of proximal extension in patients with PJK initially fused to the upper thoracic spine may result in a decreased probability of achieving optimal postoperative outcomes. Surgical invasiveness is also an important risk factor for developing PJK/PJF. Apart from the amount of levels fused, combined (anterior–posterior) surgical approaches may also be contributory. Although the combined approach frequency did not differ between both groups in this study, it has been reported in the literature to be a notable potential contributor to PJK/PJF development [4,31]. Contributors to PJK/PJF development are multifactorial, and surgical invasiveness is a risk factor, among many others [32,33]. These findings contribute to a better understanding of the outcomes associated with different levels of fusion and can guide surgeons in making informed decisions to optimize patient outcomes. These findings may also aid with informed consent, as patients may have an even more detailed understanding of the outcomes, complications, and potential risks of additional surgeries.

The present study is strengthened by its multi-center design, which ameliorates selection and expertise bias to a degree, and also the 5-year follow up period employed, which enables a longer-term assessment of postoperative outcomes. However, we also acknowledge limitations, beginning with its retrospective design. Also, the decision on UIV selection is dependent on a myriad of factors, which would be difficult to standardize in a multi-center study including multiple spine surgeons. Furthermore, hidden institutional and expertise biases may have influenced the results of this study to varying degrees. Nevertheless, we believe that the involvement of multiple institutions and surgeons contributes to the generalizability of this study’s findings. We also acknowledge that various granular surgical and non-surgical factors can contribute to postoperative outcomes, especially pertaining to implant-related complications. Although we included analyses of several surgical factors, there are potentially other impactful factors which were not included in our analysis.

## 5. Conclusions

While the risk for reoperation for proximal junctional kyphosis or failure may be lessened by primary fusion to the upper thoracic spine, such procedures may be limited by patient age, frailty, or physiology. Patients initially fused to the lower thoracic spine demonstrate an increased incidence of PJK and reoperation. Conversely, increasing levels of proximal extension in patients with PJK initially fused to the upper thoracic spine may result in a decreased probability of achieving optimal postoperative outcomes.

## Figures and Tables

**Table 1:** Baseline comparisons between UIV categories.

	UT	LT	Sig.
Age, years	61.9	65.8	0.575
BMI, kg/m^2^	27.9	28.2	0.913
ASD-mFI	3.73	3.65	0.361
CCI	1.85	2.01	0.399
Osteoporosis, %	22	15	0.009
ODI	47.3	48.1	0.322
SRS-22	2.60	2.71	0.282
EQ5D	0.72	0.74	0.746
SVA, mm	93.9	78.1	0.097
TK, °	−35.8	−31.4	<0.001
PT, °	28.5	25.3	0.968
PI-LL, °	24.7	20.7	0.229
T1PA, °	28.9	24.9	0.485

ASD-mFI = adult spine deformity modified frailty index, BMI = body mass index, CCI = Charlson Comorbidity Index, EQ5D = EuroQol 5-Item questionnaire, LT = lower thoracic group, PT = pelvic tilt, PI-LL = pelvic incidence–lumbar lordosis mismatch, ODI = Oswestry Disability Index, SRS-22 = Scoliosis Research Society-22 item score, SVA sagittal vertical axis, TK = thoracic kyphosis, T1PA = T1 pelvic angle, UT = upper thoracic group.

**Table 2:** Surgical factor and postoperative outcomes comparisons.

	UT	LT	Sig.
EBL, mL	2055.9	1774.3	0.990
Operative time, mins	503.5	416.7	0.012
LOS, days	9.6	7.7	0.024
Levels fused (posterior)	12.4	7.8	0.031
Levels fused (anterior)	1.3	1.3	0.787
Osteotomy, %	72.6	74.3	0.058
Three-column osteotomy, %	11.9	10.1	0.149
Interbody fusion levels	2.1	2.1	0.290
Implant failure, %	5	29	<0.001
Reoperations, %	38	56	0.007
W6 ODI	48.3	47.9	0.050
Y1 ODI	30.7	28.4	0.244
Y2 ODI	28.8	30	0.948
Y5 ODI	34.2	36.7	0.554
W6 SRS-22	3.02	3.12	0.276
Y1 SRS-22	3.59	3.69	0.508
Y2 SRS-22	3.64	3.64	0.602
Y5 SRS-22	3.50	3.38	0.245
W6 EQ5D	0.70	0.76	0.089
Y1 EQ5D	0.79	0.81	0.437
Y2 EQ5D	0.79	0.80	0.223
Y5 EQ5D	0.79	0.77	0.680

EBL = estimated blood loss, EQ5D = EuroQol 5-Item questionnaire, LOS = length of hospital stay, LT = lower thoracic group, ODI = Oswestry Disability Index, SRS-22 = Scoliosis Research Society-22 item score, UT = upper thoracic, W6 = Week 6, Y1 = Year 1, Y2 = Year 2, Y5 = Year 5.

## Data Availability

The original contributions presented in the study are included in the article, further inquiries can be directed to the corresponding authors.

## References

[1] Benn L., Yamout T., Tavares M.C.M., Denasty A., Blakemore L.C., Hu S.S., Hammouri Q., Minchew J., Karikari I., Osorio J. (2024). Healthcare disparities in adult and pediatric spinal deformity: A state of the art review. Spine Deform..

[2] Lee J., Park Y.S. (2016). Proximal Junctional Kyphosis: Diagnosis, Pathogenesis, and Treatment. Asian Spine J..

[3] Hyun S.J., Lee B.H., Park J.H., Kim K.-J., Jahng T.-A., Kim H.-J. (2017). Proximal Junctional Kyphosis and Proximal Junctional Failure Following Adult Spinal Deformity Surgery. Korean J. Spine..

[4] Lee B.J., Bae S.S., Choi H.Y., Park J.H., Hyun S.-J., Jo D.J., Cho Y., Korean Spinal Deformity Society (KSDS) (2023). Proximal Junctional Kyphosis or Failure After Adult Spinal Deformity Surgery—Review of Risk Factors and Its Prevention. Neurospine.

[5] Hou X., Sun Z., Li W., Wang H., Zhuo L., Yuan L., Zeng Y., Ding L. (2023). Upper instrumented vertebrae selection criteria for degenerative lumbar scoliosis based on the hounsfield unit asymmetry of the first coronal reverse vertebrae: An observational study. J. Orthop. Surg. Res..

[6] Baghdadi S., Cahill P., Anari J., Flynn J.M., Upasani V., Bachmann K., Jain A., Baldwin K., on behalf of the Harms Study Group (2021). Evidence Behind Upper Instrumented Vertebra Selection in Adolescent Idiopathic Scoliosis: A Systematic and Critical Analysis Review. JBJS Rev..

[7] Sudo H., Kaneda K., Shono Y., Iwasaki N. (2016). Selection of the upper vertebra to be instrumented in the treatment of thoracolumbar and lumbar adolescent idiopathic scoliosis by anterior correction and fusion surgery using dual-rod instrumentation: A minimum 12-year follow-up study. Spine J..

[8] Saifi C., Kang D.G., Lehman R.A. (2016). Decision making for upper instrumented vertebra in thoracolumbar/lumbar adolescent idiopathic scoliosis: Can we stop below the end vertebra?. Spine J..

[9] Ketenci I.E., Yanik H.S., Erdem S. (2018). The effect of upper instrumented vertebra level on cervical sagittal alignment in Lenke 1 adolescent idiopathic scoliosis. Orthop. Traumatol. Surg. Res..

[10] Lee J.W., Kim H.C., Kim S.I., Min H.-K., Ha K.-Y., Park H.-Y., Cho C.-H., Sung H.-S., Lim J.-H., Kim Y.-H. (2023). Effects of bone cement augmentation for uppermost instrumented vertebra on adjacent disc segment degeneration in lumbar fusions. World Neurosurg..

[11] Chen J., Chanbour H., Johnson G., Metcalf T., Lyons A., Younus I., Roth S., Stephens B., Zuckerman S. (2023). P106. Selecting the upper instrumented vertebrae in ASD surgery: What happens when you stop in the mid-thoracic spine?. Spine J..

[12] Diebo B.G., Balmaceno-Criss M., Lafage R., Daher M., Singh M., Hamilton D.K., Smith J.S., Eastlack R.K., Fessler R., Gum J.L. (2024). Lumbar Lordosis Redistribution and Segmental Correction in Adult Spinal Deformity (ASD): Does it Matter?. Spine.

[13] Passias P.G., Bortz C.A., Pierce K.E., Alas H., Brown A., Vasquez-Montes D., Naessig S., Ahmad W., Diebo B.G., Raman T. (2020). A Simpler, Modified Frailty Index Weighted by Complication Occurrence Correlates to Pain and Disability for Adult Spinal Deformity Patients. Int. J. Spine Surg..

[14] Rillardon L., Levassor N., Guigui P., Wodecki P., Cardinne L., Templier A., Skalli W. (2003). Validation of a tool to measure pelvic and spinal parameters of sagittal balance [Validation d’un outil de mesure des paramètres pelviens et rachidiens de l’équilibre sagittal du rachis]. Rev. Chir. Orthop. Reparatrice Appar. Mot..

[15] O’Brien M.F.K.T., Blanke K.M., Lenke L.G. Spinal Deformity Study Group Radiographic Measurement Manual. Medtronic Sofamor Danek USA, Inc.. https://www.oref.org/docs/default-source/default-document-library/sdsg-radiographic-measuremnt-manual.pdf?sfvrsn=2&sfvrsn=2.

[16] Champain S., Benchikh K., Nogier A., Mazel C., Guise J.D., Skalli W. (2006). Validation of new clinical quantitative analysis software applicable in spine orthopaedic studies. Eur. Spine J..

[17] Borkar S.A., Sharma R., Mansoori N., Sinha S., Kale S.S. (2019). Spinopelvic parameters in patients with lumbar degenerative disc disease, spondylolisthesis, and failed back syndrome: Comparison vis-à-vis normal asymptomatic population and treatment implications. J. Craniovertebr Junction Spine..

[18] Schwab F., Ungar B., Blondel B., Buchowski J., Coe J., Deinlein D., DeWald C., Mehdian H., Shaffrey C., Tribus C. (2012). Scoliosis Research Society-Schwab adult spinal deformity classification: A validation study. Spine.

[19] Roussouly P., Gollogly S., Berthonnaud E., Dimnet J. (2005). Classification of the normal variation in the sagittal alignment of the human lumbar spine and pelvis in the standing position. Spine.

[20] Laouissat F., Sebaaly A., Gehrchen M., Roussouly P. (2018). Classification of normal sagittal spine alignment: Refounding the Roussouly classification. Eur. Spine J..

[21] Sebaaly A., Gehrchen M., Silvestre C., Kharrat K., Bari T.J., Kreichati G., Rizkallah M., Roussouly P. (2020). Mechanical complications in adult spinal deformity and the effect of restoring the spinal shapes according to the Roussouly classification: A multicentric study. Eur. Spine J..

[22] Lafage R., Smith J.S., Elysee J., Passias P., Bess S., Klineberg E., Kim H.J., Shaffrey C., Burton D., Hostin R. (2022). Sagittal age-adjusted score (SAAS) for adult spinal deformity (ASD) more effectively predicts surgical outcomes and proximal junctional kyphosis than previous classifications. Spine Deform..

[23] Lovecchio F., Lafage R., Line B., Bess S., Shaffrey C., Kim H.J., Ames C., Burton D., Gupta M., Smith J.S. (2023). Optimizing the Definition of Proximal Junctional Kyphosis: A Sensitivity Analysis. Spine.

[24] Yoshida G., Hasegawa T., Yamato Y., Kobayashi S., Shin O., Banno T., Mihara Y., Arima H., Ushirozako H., Yasuda T. (2019). Minimum Clinically Important Differences in Oswestry Disability Index Domains and Their Impact on Adult Spinal Deformity Surgery. Asian Spine J..

[25] Daniels A.H., Reid D.B.C., Durand W.M., Hamilton D.K., Passias P.G., Kim H.J., Protopsaltis T.S., Lafage V., Smith J.S., Shaffrey C.I. (2019). Upper-thoracic versus lower-thoracic upper instrumented vertebra in adult spinal deformity patients undergoing fusion to the pelvis: Surgical decision-making and patient outcomes. J. Neurosurg. Spine.

[26] Fujimori T., Inoue S., Le H., Schairer W.W., Berven S.H., Tay B.K., Deviren V., Burch S., Iwasaki M., Hu S.S. (2014). Long fusion from sacrum to thoracic spine for adult spinal deformity with sagittal imbalance: Upper versus lower thoracic spine as site of upper instrumented vertebra. Neurosurg. Focus.

[27] Luo M., Wang P., Wang W., Shen M., Xu G., Xia L. (2017). Upper Thoracic versus Lower Thoracic as Site of Upper Instrumented Vertebrae for Long Fusion Surgery in Adult Spinal Deformity: A Meta-Analysis of Proximal Junctional Kyphosis. World Neurosurg..

[28] Fu X., Sun X.L., Harris J.A., Sheng S.-R., Xu H.-Z., Chi Y.-L., Wu A.-M. (2016). Long fusion correction of degenerative adult spinal deformity and the selection of the upper or lower thoracic region as the site of proximal instrumentation: A systematic review and meta-analysis. BMJ Open.

[29] Chen J., Shao X.X., Sui W.Y., Yang J.-F., Deng Y.-L., Xu J., Huang Z.-F., Yang J.-L. (2020). Risk factors for neurological complications in severe and rigid spinal deformity correction of 177 cases. BMC Neurol..

[30] Cammarata M., Aubin C.É., Wang X., Jean-Marc M.-T. (2014). Biomechanical risk factors for proximal junctional kyphosis: A detailed numerical analysis of surgical instrumentation variables. Spine.

[31] Kim H.J., Yang J.H., Chang D.G., Suk S.-I., Suh S.W., Kim S.-I., Song K.-S., Park J.-B., Cho W. (2022). Proximal Junctional Kyphosis in Adult Spinal Deformity: Definition, Classification, Risk Factors, and Prevention Strategies. Asian Spine J..

[32] Nguyen N.L., Kong C.Y., Hart R.A. (2016). Proximal junctional kyphosis and failure-diagnosis, prevention, and treatment. Curr. Rev. Musculoskelet. Med..

[33] Zhu W.Y., Zang L., Li J., Guan L., Hai Y. (2019). A biomechanical study on proximal junctional kyphosis following long-segment posterior spinal fusion. Braz. J. Med. Biol. Res..

